# Greener Synthesis of Zinc Oxide Nanoparticles: Characterization and Multifaceted Applications

**DOI:** 10.3390/molecules25184198

**Published:** 2020-09-14

**Authors:** Ali Aldalbahi, Seham Alterary, Ruba Ali Abdullrahman Almoghim, Manal A. Awad, Noura S. Aldosari, Shouq Fahad Alghannam, Alhanouf Nasser Alabdan, Shaden Alharbi, Budur Ali Mohammed Alateeq, Atheer Abdulrahman Al Mohsen, Munirah A. Alkathiri, Raghad Abdulrahman Alrashed

**Affiliations:** 1 Department of Chemistry, College of Science, King Saud University, Riyadh 11451, Saudi Arabia; salterary@ksu.edu.sa; 2 King Abdullah Institute of Nanotechnology, King Saud University, Riyadh 11451, Saudi Arabia; 3 Department of Biochemistry, College of Sciences, King Saud University, Riyadh 11451, Saudi Arabia; Rubaalmoghim@gmail.com (R.A.A.A.); Alhanoufalabdan@gmail.com (A.N.A.); 4 Department of Botany and Microbiology, College of Science, King Saud University, Riyadh 11451, Saudi Arabia; nsaldosari@ksu.edu.sa (N.S.A.); Shouq.alghnnam@gmail.com (S.F.A.); Bedoor.mic@gmail.com (B.A.M.A.); atheer.ab.almohsen@gmail.com (A.A.A.M.); 5 Department of Clinical Laboratory Sciences, College of Applied Medical Sciences, King Saud University, Riyadh 11451, Saudi Arabia; shadenalharbi1@gmail.com; 6 Department of Clinical Pharmacy, College of Pharmacy, King Saud University, P.O. Box 2457, Riyadh 11451, Saudi Arabia; Munirah.Alkathiri@hotmail.com (M.A.A.); Raghad.alrashedd@gmail.com (R.A.A.)

**Keywords:** *K. blossfeldiana*, green synthesis, ZnO nanoparticles, photoluminescence, transmission electron microscopy, X-ray diffraction, photodegradation, antimicrobial and cytotoxicity potential

## Abstract

Nanoparticles (NPs) have unique properties compared to their bulk counterparts, and they have potentials for various applications in many fields of life science. Green-synthesized NPs have garnered considerable interest due to their inherent features such as rapidity, eco-friendliness and cost-effectiveness. Zinc oxide nanoparticles (ZnO NPs) were synthesized using an aqueous extract of *Kalanchoe blossfeldiana* as a reducing agent. The resulting nanoparticles were characterized via X-ray diffraction (XRD), dynamic light scattering (DLS), UV-Vis spectroscopy, photoluminescence (PL), transmission electron microscopy (TEM), scanning electron microscopy (SEM) and energy-dispersive spectroscopy (EDS). The antimicrobial potential of the synthesized ZnO NPs against bacterial and fungal strains was examined by the disk diffusion method, and they showed a promising antibacterial and antifungal potential. The catalytic activity of the synthesized ZnO NPs in reducing methylene blue (MB) and eosin was studied via UV-Vis spectroscopy. The decolorization percentages of the MB and Eosin Y dyes were 84% and 94%, respectively, which indicate an efficient degradation of the ZnO NPs. In addition, the cytotoxic activity of the ZnO NPs on the HeLa cell line was evaluated via in vitro assay. The MTT assay results demonstrate a potent cytotoxic effect of the ZnO NPs against the HeLa cancer cell line.

## 1. Introduction

Nanotechnology is one of the fastest-growing technologies, which will likely form the basis of multiple technological and biotechnological innovations; hence, it is considered as the upcoming industrial revolution of the century [1]. Nanotechnology has been employed in various academic and industrial fields, including chemistry, biology, medicine and physics [2,3,4]. Owing to their superior physicochemical and biological properties over the bulk materials, nanomaterials have significant potential in different fields of science [5]. The size of nanoparticles (NPs) (1–100 nm) offers a higher surface-to-volume ratio, which results in high surface reactivity [6,7]. This distinct property makes NPs extensively useful in many fields (e.g., catalysis, gas sensing, electronics and environmental remediation) and promotes their integration into various commercial products, biotechnology and biomedical applications [8].

Nanobiotechnology represents the merging of biotechnology and nanotechnology to develop green, synthetic and eco-friendly technology for the syntheses of nanomaterials [9,10]. The progress in the use of metal oxide nanoparticles in environmental applications has stimulated the requirement for their synthesis using green chemistry techniques via biological systems that account for environmental aspects [11,12]. Metal oxides have remarkable properties such as optimal chemical stability, solubility and adhesiveness. The reaction of a salt or ligand has broadened the spectra of many biological and environmental research areas and plays a significant role in electronic devices, radiolabeling, medicinal applications and contaminant detection [13].

Zinc oxide is the most promising inorganic oxides that are extensively used owing to its suitable magnetic, electrical and optical properties. ZnO, as a semiconductor, is abundant in its three forms: hexagonal wurtzite, rock salt and cubic structures [14]. Among them, wurtzite has a direct bandgap energy of 3.40 EV [15]; thus, it is transparent in the visible region with most activity occurring in the UV/blue region. Moreover, the conductivity and luminescence of ZnO enhance the optical absorption and UV filtering property, resulting in an advanced biosensing ability of ZnO NPs [16]. ZnO is non-toxic and its NPs possess a high surface-to-volume ratio. Owing to the unique electrical, chemical and optical properties of ZnO NPs, they are used in wide applications (e.g., solar cells, light-emitting diodes, metal-insulator-semiconductor diodes, photocatalytic degradation, drug delivery and personal care products such as cosmetics and sunscreens) [17,18], which make them the most feasible, economically viable and time-efficient approach as the chemicals are less hazardous to the environment.

In the synthesis of nanomaterials, the use of biological components has always been a preferred choice for an environmentally friendly approach known as green synthesis. In addition to the environmental ecosystem benefits, green synthesis has been proved to be very useful in controlling the desired NP size and shape [19]. Various studies have suggested that plant extracts are better candidates and more appropriate for large-scale green NP synthesis owing to the high rate of synthesis compared to when other organisms are used. NPs synthesized from plants have more variations in shape and size compared with those synthesized from other organisms such as bacteria, fungi and yeast [20,21].

This paper reports the green synthesis of ZnO NPs from an aqueous extract of *K. blossfeldiana* and zinc nitrate hexahydrate as the Zn precursor. The formation of ZnO NPs was examined and characterized by different spectroscopic and microscopic techniques, including XRD, UV-Vis spectroscopy, PL, TEM, SEM and EDS. The objective of this study was to screen the activity of the synthesized eco-friendly ZnO NP against different pathogen strains and evaluate their cytotoxic effects against the HeLa cell line by MTT assay. In addition, the structural, microscopic, spectroscopic and photocatalytic degradation activity of toxic dyes such as methylene blue (MB) and Eosin Y were investigated. To our knowledge, this is the first report on an aqueous *K. blossfeldiana* extract-mediated synthesis of ZnO NPs [22].

## 2. Results and Discussion

### 2.1. Nanoparticles Characterization Results

**Figure 1:**
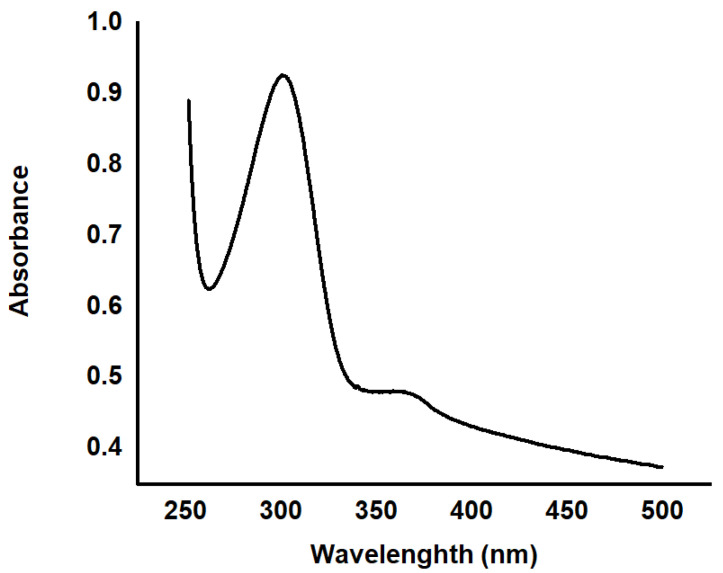
Absorption spectra of the synthesized ZnO NPs.

The UV-Vis spectrophotometer was used to characterize the structural properties of the NPs by determining the absorbance, confirming the formation of previous findings and verifying that the *K. blossfeldiana* extract yielded a stable ZnO NP synthesis. Figure 1 shows the UV-Vis absorption spectra of the ZnO NPs observed at 200–600 nm. The peaks of the excitonic absorption were between 300 and 400 nm, which are typical characteristic ZnO NPs peaks, thus, confirm their presence. This may be attributed to the electron transition from the valence to the conduction band. An absorption peak was also recorded at ~300 nm corresponding to the ZnO NPs that lie considerably below the bandgap wavelength of 359 nm.

The maximum absorbance was observed at 300 and 359 nm, and the corresponding bandgap (*E_g_*) was calculated using the formula:

(1) Eg=1240λ

The corresponding bandgap energies were 4.13 and 3.45 eV, respectively. As the particle size decreased, the energy gap increased as a result of the quantum size effects on the electronic energy bands of semiconductors. This becomes more prominent when the size of the nano-crystallites is less than the Bohr radius. The coulombic interactions between holes and electrons play a crucial role in nano-sized solids. The quantum confinement of charge carriers modifies the valence and conduction bands of semiconductors [23,24]. The considerably sharp absorption peaks of ZnO confirms the monodispersed nature of the NP distribution [25,26].

**Figure 2:**
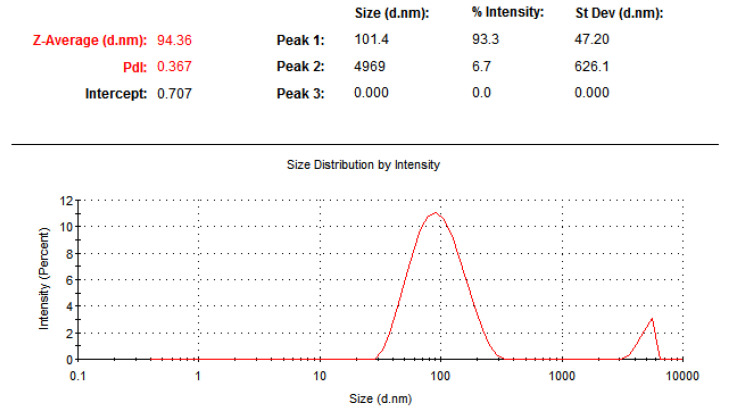
DLS measurements results of the synthesized ZnO NPs.

The DLS technique was used to determine the mean Z-average diameter (nm) of the particles and the size distribution of the resulting ZnO NPs. As shown in Figure 2, the mean average size of the NPs was about 94 nm. The size distribution profile of the ZnO NPs showed two significant peaks with intensities of 93.3% and 6.7%. The polydispersity index of the ZnO NPs was 0.367, which indicates that the synthesized particles had a little agglomeration and variation in size [27]. DLS is known to provide considerably larger values than TEM size analysis, possibly because of the hydrodynamical shell. The size of the hydrodynamical shell depends on the particle structure, particle shape and roughness [28].

**Figure 3:**
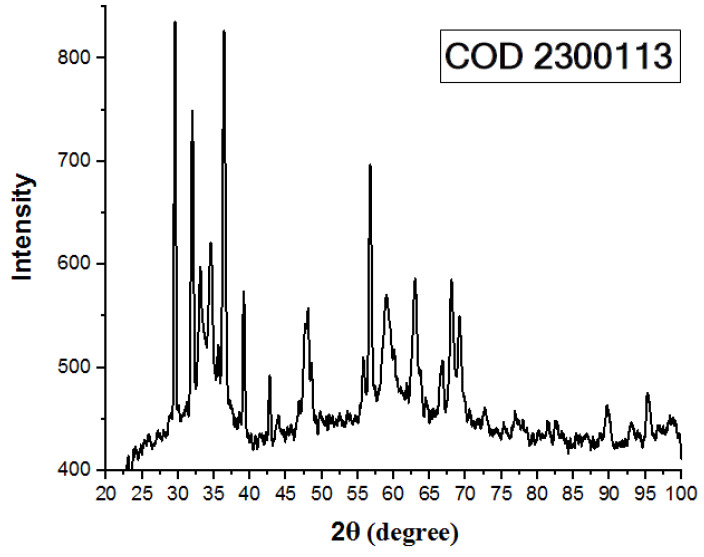
XRD analysis of the synthesized ZnO NPs.

The XRD pattern of the synthesized ZnO NPs is shown in Figure 3. The broad peaks indicate that the synthesized material has particles in the nanoscale range [26]. The 2θ angles of the diffraction peaks were located at ~31.926°, 34.624°, 36.442°, 47.808°, 56.894°, 63.242°, 66.742°, 68.340° and 69.471° for the (100), (002), (101), (102), (210), (103), (200), (212) and (201) planes, respectively. These peaks are consistent with those reported in [29] and are indexed as the hexagonal wurtzite phase of ZnO NPs [30] with lattice constants a = b = 0.323420 nm and c = 0.517720 nm (COD 2300113) [31]. Moreover, the XRD spectrum showed no peaks other than the characteristic ZnO peaks, which confirms that the synthesized ZnO NPs were pure. Furthermore, the narrow and robust diffraction peak indicates an optimum crystalline structure of ZnO NPs [29,32].

**Figure 4:**
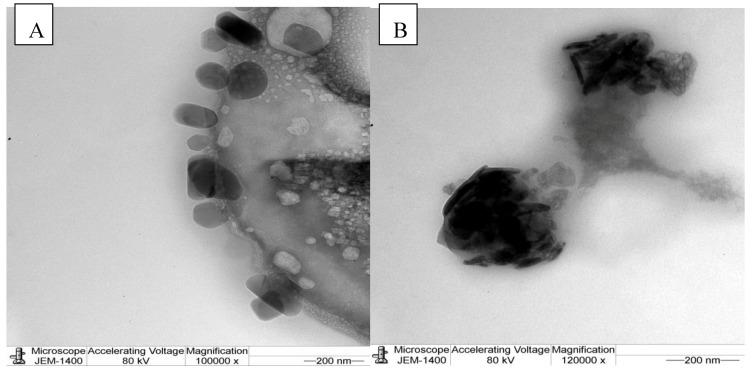
TEM images of the synthesized ZnO NPs, (**A**) spherical structure and (**B**) rod-shaped structure.

The size and shapes of the NPs were studied using TEM. Figure 4A,B shows the images of the different shapes of the synthesized ZnO NPs. The morphology of the NPs showed hexagonal, spherical (Figure 4A) and rod-shaped (Figure 4B) structures with some agglomerated large and small particles.

**Figure 5:**
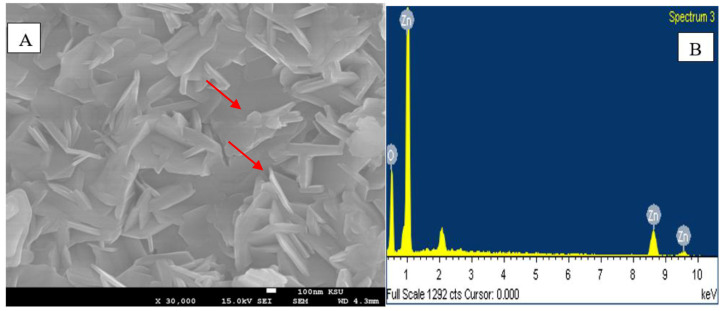
(**A**) SEM image; and (**B**) EDS analysis of the synthesized ZnO NPs.

The surface morphology and size of the green-synthesized ZnO NPs were imaged using SEM, and the chemical composition was determined by EDX (Figure 5A,B). Most of the ZnO NPs were on the nanometer scale and in rods, hexagonal or other various shapes (Figure 5A), which was confirmed via TEM analysis. Remarkably, the largest amounts of ZnO NPs were identical in dimension with a small amount of large particles. The ZnO NPs also showed some agglomeration, which is typical with a green synthesis of NPs. This agglomeration is attributed to the fact that green-synthesized NPs possess a higher surface area and they strongly stick to each other [33]. Therefore, ecological factors highly influence the stability of NPs and their agglomeration. During NP formation processes, the NPs have a considerable affinity to each other and they form asymmetrical clusters [34]. The EDX spectrum shows strong major peaks between 0.5 and 9.6 keV, which confirms the elemental distribution of the ZnO NPs [35]. The EDX characterization revealed that the ZnO powder is a high-purity compound [36] with high Zn and oxygen contents, as shown in Figure 5B.

**Figure 6:**
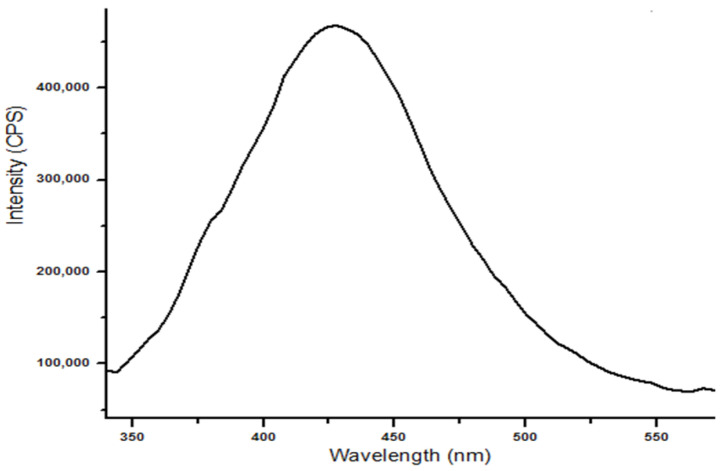
PL spectrum of the synthesized ZnO NPs.

The PL spectrum of the synthesized ZnO NPs was measured at room temperature. At the excitation peak at 300 nm and near their band edge, an emission peak at 430 nm was recorded (Figure 6). The ZnO surface defects, which are most likely oxygen deficiency, gave rise to the defect emission in the visible region. In addition, similar results were obtained by Jangir who reported that the emission spectrum shows a broad yellow emission at 570 nm along with sharp peaks at ~400 and ~460 nm corresponding to pure ZnO, which indicates the presence of a large number of surface defects (singly ionized oxygen vacancies) [37]. Moreover, the transition between the single-charged oxygen vacancies and the photoexcited holes in the valence band of the ZnO NPs gave rise to the broad peak [38]. The quantum size effect dominates when the particle size reaches a nanometer scale. This alters the physical properties of the semiconductor materials. PL originates from the recombination of surface states and sharp and strong PL represents very shallow surface states. The shallow trap centers due to the quantum yields of the band edge may capture photoexcited electrons and transfer the carriers to the surface for the photocatalytic process [39,40].

### 2.2. Photocatalytic Degradation of Dyes

**Figure 7:**
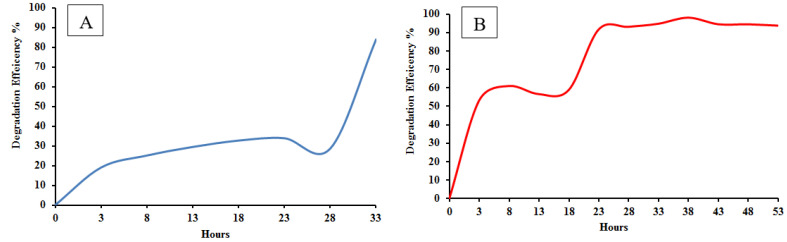
(**A**) MB dye; and (**B**) EosinY dye degradation efficiency of the synthesized ZnO NPs.

The photocatalytic activities of the ZnO NPs were estimated by monitoring the photodegradation of MB and Eosin dyes in aqueous solutions under UV radiation. The rate of degradation was determined by recording the reduction in the absorption intensity of the dyes at the maximum wavelength using the UV-Vis spectrophotometer. The solutions of the dyes were loaded with a photocatalyst, and their optical absorption spectra were observed over different durations of light exposure. Figure 7 shows the photocatalytic activities of the ZnO NPs. The green ZnO NPs catalysts showed a good response under UV irradiation. The color and concentration of the dyes were visually observed to gradually decrease with the photocatalysis time. The characteristic absorption maxima of the MB dye at ~664 and 291 nm disappeared almost completely after 33 h of photocatalysis (Figure 7A), whereas that of the Eosin Y dye at ~515 and 291 nm disappeared after 53 h (Figure 7B), which indicates an efficient degradation of the synthesized ZnO NPs. The percentage of decolorization of the MB and Eosin Y dyes were 84% and 94%, respectively (Figure 7A,B). The light intensity and electron–hole formation in the photochemical reaction were responsible for initiating the rate, as reported in [41].

**Scheme 1:**
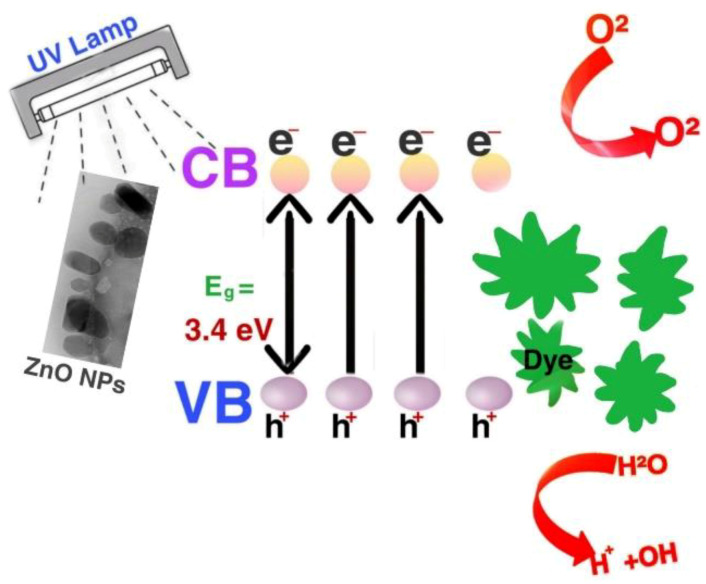
Illustration of the mechanism of the photocatalytic degradation of the synthesized ZnO NPs.

Scheme 1 shows the well-known basic mechanism of photocatalytic activity of ZnO NPs [42]. On illuminating the suspension with UV light, the ZNO NPs are irradiated and the electrons jump from the valence band (VB) to the conduction band (CB). This generates electron–hole pairs, which form free radicals also called activated oxygen species (AOS) on reaction with water. These free radicals oxidize the biomolecules (organic pollutants) leading to the degradation of the MB and Eosin Y dyes. The band gap value of bulk ZnO is 3.3 eV [43], which causes a swift recombination of the electrons and holes, leading to ineffective degradation of the biomolecules. The ZNO NPs showed a lower the band gap value than 3.3 eV. The green synthesis plays a vital role here as the plant extracts used act as a capping agent for the ZnO NPs, which stabilizes them, and further enhances charge separation. This prevents the recombination of electrons (e^−^) and holes (h^+^), which in turn increases the formation of AOS, making the degradation process of the dye more efficacious. [44,45].

### 2.3. Antimicrobial Assessment Results

**Figure 8:**
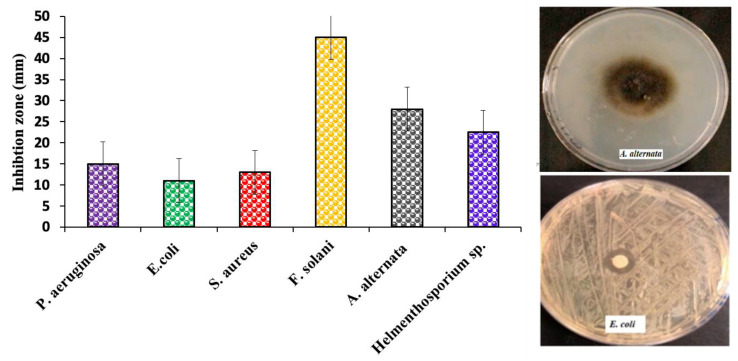
Antimicrobial effects of the synthesized ZnO NPs against several pathogens.

Antimicrobial activity of the ZnO NPs synthesized from *K. Blossfeldiana* aqueous extract was quantitatively evaluated on the basis of the inhibition zones using the agar disc diffusion method against different organisms: *S. aureus* (Gram-positive bacteria); *E. coli* and *P. aeruginosa* (Gram-negative bacteria); and *Fusarium solani*, *Alternaria alternate* and *Helmenthosporium sp.* fungi. The results confirm that the ZnO NPs exhibit significant antibacterial activity on all bacterial strains. *P. aeruginosa* recorded the highest inhibition zone (15 mm), followed by *S. Areas* (13 mm) and *E. Coli* (11 mm). On the other hand, *F. Salami* showed the maximum inhibition zone among the fungi (45 mm), followed by *A. Alternate* (28 mm) and *Helmenthosporium sp.* (22.5 mm), as shown in Figure 8.

Zaire et al. reported that *P. aeruginosa* is more sensitive to ZnO NPs than *S. aureus*, and the resistance of *S. aureus* could be attributed to the presence of a thick layer of peptidoglycan in its cell walls [46]. However, herein, *E. coli* exhibited higher resistance than *S. aureus*. This can be attributed to the difference in the polarity of the cell membrane. This is because the membrane of *S. aureus* has a lower negative charge than that of *E. coli*, which in turn promotes the penetration of high levels of negatively charged free radicals into *S. aureus* and thus leads to its death [47,48,49].

The electrostatic interactions that form at the interface facilitate the penetration of ZnO NPs into the microbial cell membrane, which would result in cell damage through the release of reactive oxygen species (ROS) from microbes. H_2_O_2_ is produced from the ZnO surfaces and distributed all over the cell; thus, the fungi or bacteria are exterminated [50,51].

### 2.4. In Vitro Cytotoxicity Assay Results

**Figure 9:**
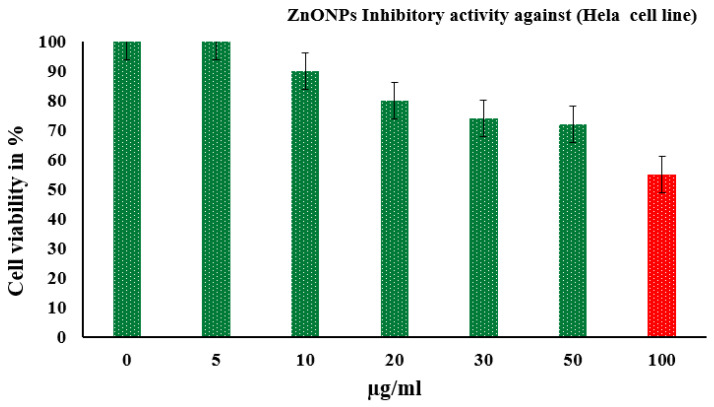
Results of the in vitro cytotoxicity assay of the synthesized ZnO NPs on HeLa cells.

The in vitro cytotoxic activity of the green-synthesized ZnO NPs against HeLa cell lines was evaluated using MTT assay at different concentrations. The ZnO NPs inhibited the reproduction of cells in a dose-dependent manner. Figure 9 shows the percentage cell viability of the carcinoma HeLa cell lines examined using the MTT colorimetric assay after 24 h of exposure to six concentrations (5–100 µg L^−1^) of ZnO NPs. The results show that the treatment with the ZnO NPs significantly inhibited the growth of cells (*p* ≤ 0.05) and the reduction was concentration dependent.

The inhibitory concentration of the ZnO NPs was ~100 µg L^−1^. The specific properties of the ZnO NPs, including biocompatibility, green synthesis excessive selectivity and improved cytotoxicity, make the NPs a promising anti-cancer agent [52,53]. The cytotoxic activity of metal oxide NPs has been shown to depend on their shape, size and capping agents [54]. Some previous studies reported high cytotoxicity for ZnO NPs, while others demonstrated no toxic effect. This variation could be attributed to a difference in the amount of ZnO NPs or changes in cell density [55]. The results obtained herein are consistent with the previous studies that reported significant cytotoxicity of ZnO NPs to human hepatocellular carcinoma *HepG2* [56,57], bronchial epithelial *BEAS-2B*, lung adenocarcinoma *A549* [58], colon adenocarcinoma *Caco-2* [20], breast *MCF-7* and *MDA* [59] cancer cells.

The exact cytotoxic mechanism of ZnO NPs is yet to be established; thus, several mechanisms have been proposed. One of them suggests that the main mechanism of ZnO NPs cytotoxicity is the intracellular release of dissolved zinc ions, accompanied by the induction of ROS. This action is induced by a binary response comprising of the pro-inflammatory reaction of the cells against ZnO and the characterization of the surface properties of the NPs (semiconductor nature), where the electrons leap to the CB [60]. Electrons and holes frequently and rapidly recombine and move to the surface of the NPs where they react with the adsorbed species [61]. This results in a change in the surface of the NPs by elevating a variety of electrons and holes, followed by the generation of ROS and oxidative stress, which eventually cause the death of cells. However, the anti-oxidative capability of the cell is surpassed; thus, ZnO NPs would act as a redox system [20,62].

## 3. Materials and Methods

### 3.1. Preparation of K. Blossfeldiana Extract

A wilting house plant *K. blossfeldiana* was obtained from a local market in Riyadh, Saudi Arabia, cut into small pieces and washed properly. Boiled distilled water (100 mL) was added to 52 g of the *K. blossfeldiana* pieces and homogenized using a blender (Waring, 8011S, Chula Vista, CA, USA). Then, the mixture was filtered through a gauze and centrifuged at 5000 rpm for 10 min to form an extract. The supernatant was then filtered to obtain the extract, which was then stored at 4 °C.

### 3.2. Green Synthesis of ZnO NPs

Zinc nitrate hexahydrate (0.5 M; Zn (NO_3_)_2_·6H_2_O, Lobachemie, Maharashtra, India) was dissolved in 50 mL of the *K. blossfeldiana* extract to form a mixture and stirred at 90 °C. An aqueous solution of 1 M sodium hydroxide (NaOH, Qualikems Fine Chem Pvt. Ltd., Vadodara, India) was then added dropwise into the mixture and stirred using a magnetic stirrer. Stirring continued for 30 min after the addition of NaOH. The resulting precipitates were dried at 200 °C for 5 h to form a beige powder containing the ZnO NPs. Then, the powder, which had a beige color, was calcined in a muffle furnace (CWF, 1300, Carbolite, Derbyshire, UK) at 600 °C for 4 h. A white powder containing zinc oxide nanoparticles was obtained.

### 3.3. Characterization of ZnO NPs

The optical properties of the green-synthesized ZnO NPs were investigated using a UV-1800 UV-Vis spectrometer (Shimadzu, Reinach, Switzerland) in the range of 200–600 nm, and the emission spectrum was measured using a Fluorolog 3 spectrofluorometer (FL-3-11, Horiba JobinYvon, Piscataway, NJ, USA). The particle size was determined via dynamic light scattering (DLS) technique using a Zetasizer (HT Laser, ZEN3600 Malvern Instruments, Malvern, UK). The crystalline structures of the particles were investigated using a Bruker D8 ADVANCE X-ray diffractometer (Bruker, Billerica, MA, USA) operating at 40 KV and 40 MA with CuKa radiation at 1.5418 Å. The surface morphology and sizes of the NPs were studied by TEM with an acceleration voltage of 200 KV (JEM-2100F, JEOL Ltd., Dearborn, MI, USA). An energy-dispersive X-ray spectrometer (EDS, Oxford Instruments, High Wycombe, UK) attached to the SEM (FESEM, JEOL, Albuquerque, NM, USA) was used to identify and map the elements present in the ZnO NP powder.

### 3.4. Photocatalytic Analysis Measurements

Under UV irradiation, the photocatalytic activity of the green-synthesized ZnO NPs was examined with MB and Eosin Y dyes (Sigma-Aldrich, St. Louis, MO, USA). A 30-mL dye solution was placed in a laboratory-scale cuvette and photocatalyst samples were dispersed within the cuvette to face the UV light at a distance from the UV lamp. To monitor the degradation rate for the MB and Eosin dyes, the optical absorption spectra were determined at different durations of light exposure using the UV-Vis spectrophotometer (Shimadzu, Reinach, Switzerland). This was achieved by recording the reduction in the absorption intensity of the dyes at the maximum wavelength. Then, the degradation efficiency (*DE*) was calculated using the following equation:

(A_0_ − A)/A_0_ × 100 = *DE*%, (2)

A_0_ is the initial absorption and A is the absorption intensity after photodegradation. The entire reduction reaction experiment was conducted under UV irradiation. The intensity of the blue (MB) and red (Eosin) colors and the reaction mixtures gradually decreased and completely became colorless. The dye absorbance was examined using the UV-Vis spectrometer (Shimadzu, Reinach, Switzerland) as an indicator of the catalytic activity of the ZnO particles. The possible mechanism [23] is given by the following equations:

(3) $$\mathrm{Greener}\;\mathrm{ZnO}\;\mathrm{NPs}\;+\;h\nu \to e_{\mathrm{CB}}^{-}+h_{\mathrm{VB}}^{+}$$

(4) $$h_{\mathrm{VB}}^{+}+{OH}^{-\;}\to {OH}^{ࢫ}$$

(5) $$H_{2}O+h_{\mathrm{VB}}^{+}\to {OH}^{∙}+H^{+}$$

(6) $$e_{\mathrm{CB}}^{-}+O_{2}\to O_{2}^{∙}$$

(7) $$O_{2}^{∙}+e_{\mathrm{CB}}^{-}+{2H}^{+}\to H_{2}O_{2}$$

(8) $${2H}^{+}+{2O}_{2}^{∙}\to H_{2}O_{2}+O_{2}$$

(9) $$e_{\mathrm{CB}}^{-}+H_{2}O_{2}\to {OH}^{∙}+{OH}^{-}$$

(10) $$O_{2}^{∙}+\mathrm{Dyes}\to \mathrm{Oxidation}\;\mathrm{products}\to \mathrm{Final}\;\mathrm{species}$$

(11) $${OH}^{∙}+\mathrm{Dyes}\to \mathrm{Oxidation}\;\mathrm{products}\to \mathrm{Final}\;\mathrm{species}$$

where ($e_{\mathrm{CB}}^{-}$) is a photoelectron and $\left(h_{\mathrm{VB}}^{+}\right)$ is a photoinduced holes.

### 3.5. Assay of Microbial Activity of ZnO NPs

The antimicrobial potential of the ZnO NPs synthesized from the *K. blossfeldiana* extract was tested using the agar well diffusion method. The human pathogenic microorganisms used in this study included four bacterial strains (*Escherichia coli*, *Pseudomonas aeruginosa* and *Staphylococcus aureus*) and three fungal strains (*Helmenthosporium* sp, *Alternaria alternate* and *Fusarium oxysporium*). The bacterial cultures were grown on blood agar at 37 °C for 18 h, and then the colonies were suspended in a saline solution (0.85% NaCl) with the turbidity adjusted to 0.5 MacFarland standard (10^8^ colony forming units (CFU/mL)). The bacterial and fungal suspensions were swabbed on Muller-Hinton agar plates and a potato dextrose agar medium, respectively. The wells were cut and 100 μg L^−1^ of the synthesized ZnO NPs were loaded in the wells. The plates were then incubated at 37 °C for 24 h (for bacteria) and 28 °C for 48–72 h (for fungi). Thereafter, the plates exhibited the formation of clear inhibition zones around the wells, which indicate the occurrence of antimicrobial activity. The inhibition zones were then calculated by measuring their diameter around the wells.

### 3.6. In Vitro Cytotoxicity Assay

Cervical carcinoma HeLa cells were characterized as an adherent and continuous cell when grown in optimized cell culture (5% CO_2_ in an air atmosphere and 37 °C). The cells were regularly checked using an inverted microscope. The total number of cells used in the different experiments was determined by the 0.4% trypan blue exclusion test. HeLa cells were seeded in a 96-well plate at a density of 2 × 10^5^ cells/well in a 90 µL Dulbecco’s Modified Eagle’s Medium. For the cytotoxicity assay, the cells were allowed to settle for a day before being treated with six different concentrations (5–100 µg L^−1^) of the green ZnO NPs. Then, the treated cells were allowed to grow further for 24 h. The experiment was further performed on four replicates. The optical densities were normalized according to a control (untreated cells). The cell viability of the untreated cells was 100% and that of the treated cells was above or below 100%. The following equation was used for the calculations.

(12) $$\mathrm{Cell}\;\mathrm{viability}\;(\%)\;=\left(\frac{\mathrm{Absorbance}\;\mathrm{of}\;\mathrm{individual}\;\mathrm{treatment}}{\mathrm{Absorbance}\;\mathrm{of}\;\mathrm{the}\;\mathrm{control}}\right)\times 100$$

## 4. Conclusions

This paper presents the synthesis of ZnO NPs via a green synthesis technique using an aqueous extract of *K. blossfeldiana*. This approach promotes the use of green synthesis for other metal NPs such as Ag and Au. The synthesized NPs showed characteristic UV-Vis absorption peaks at 359 and 300 nm. XRD analysis also revealed the hexagonal wurtzite structure. The ZnO NPs were characterized by EDS equipped with SEM, TEM and DLS, which confirmed the presence of NPs with an average size of 94.36 nm. The synthesized ZnO NPs exhibited excellent efficiency in the photocatalytic degradation of MB (84%) and Eosin Y (94%) in aqueous solutions under UV light irradiation. They also demonstrated excellent antimicrobial activities against many strains and exhibited an attractive selective cytotoxic activity against different cancer cells. ZnO NPs offer safer and cheaper alternatives to conventional therapies. Their promising characteristics and green synthesis make them a potential candidate for various environmental- and health-related applications.
